# Chitosan–Carboxymethylcellulose-Based Polyelectrolyte Complexation and Microcapsule Shell Formulation

**DOI:** 10.3390/ijms19092521

**Published:** 2018-08-25

**Authors:** Jagadish Chandra Roy, Ada Ferri, Stéphane Giraud, Guan Jinping, Fabien Salaün

**Affiliations:** 1 Departments of Applied Science and Technology, Politecnico di Torino, 10129 Torino, Italy; ada.ferri@polito.it; 2 Department of Mechanical, Energy and Material Engineering, University of Lille Nord de France, F-5900 Lille, France; stephane.giraud@ensait.fr (S.G.); fabien.salaun@ensait.fr (F.S.); 3 Department of Textile Material Engineering, École Nationale Supérieure des Arts et Industries Textiles, 59056 Roubaix, France; 4 College of Textile and Clothing Engineering, Soochow University, Suzhou 215000, China; guanjinping@suda.edu.cn

**Keywords:** chitosan, carboxymethyl cellulose sodium salt, microcapsule, emulsion, polyelectrolyte complex

## Abstract

Chitosan (CH)–carboxymethyl cellulose sodium salt (NaCMC) microcapsules containing paraffin oil were synthesized by complex formation, and crosslinked with glutaraldehyde (GTA). The electrostatic deposition of NaCMC onto the CH-coated paraffin oil emulsion droplets was demonstrated by zeta potential and optical microscopy. The optimal process conditions were identified in terms of pH of the aqueous solution (5.5) and CH/NaCMC mass ratio (1:1). Encapsulation of paraffin oil and microcapsule morphology were analyzed by ATR-FTIR and SEM, respectively. The effect of GTA crosslinking on paraffin oil latent heat was investigated by DSC and combined with the values of encapsulation efficiency and core content, supporting the compact shell formation.

## 1. Introduction

The formation of polyelectrolyte (PE) complexes (PEC) is achieved by involving two oppositely charged PEs, and is governed by intrinsic and extrinsic charge compensation. Two oppositely charged PEs take part in the intrinsic charge compensation while the counterions neutralize the PE charge in the extrinsic charge compensation. Therefore, complexation is one of most efficient techniques to form a thin film or a coating in which the desired thickness can be tuned precisely in the nanometer or micrometer scale. Film or membrane design by molecular assembly or complexation involving oppositely-charged PEs is achieved in many different ways, such as layer-by-layer technique, colloidal dispersion, and film assembly. PEC assembly is highly sensitive to pH, temperature, ionic strength, concentration of PE, etc. [1]. However, PECs were applied to many different and diverse applications, such as surgical adhesives [2], additives to reinforce paper strength [3], vectorization and drug, controlled release [4], ultrafiltration [5]. In microcapsule formulation, the PEC acts as a container by shell formation and encapsulation of drug molecules, whose release kinetics depend on the shell properties. Therefore, it is quite important to have a successful oil-in-water emulsion preparation during the initial formulation stage, in which polysaccharides and/or polyelectrolyte complexes (PEC) act as emulsifier and stabilizer. Upon crosslinking of the emulsifier in the aqueous phase, a biopolymer film is formed around the oil phase with the aim of protecting the core from the external environment, prolonging shelf-life and controlling the drug release. Therefore, a suitable choice of polysaccharide composition and optimized process conditions are crucial for the formation of polyelectrolyte complex and emulsion formulation.

Among commercial polysaccharides, chitosan (CH) is a positively charged derivative from the second most abundant biopolymer (after cellulose) produced by the fish industry as waste material—chitin [6]. Under the action of concentrated sodium hydroxide, chitin *N*-acetyl-glucosamine units are modified and deacetylated. When deacetylation exceeds 50% of the *N*-acetyl-glucosamine units, chitin starts to be soluble in the acidic medium and is called chitosan (CH). CH contains (1,4)-linked d-glucosamine and *N*-acetyl-d-glucosamine monomers distributed in the linear chain randomly. In addition, the amino groups of the d-glucosamine unit are very prone to solubilizing and possess a positive charge acidic medium when the deacetylation exceeds more than 50%. Consequently, it easily bound to negatively charged functional groups. Therefore, the degree of deacetylation (DD) and the molecular weight are both crucial parameters for CH selection. CH acts as a cationic polyelectrolyte in acidic medium where the pH is under the p*K*a of CH (below 6.4) and, being non-toxic to human body and the environment, it is biocompatible and biodegradable [7]. CH acts as an emulsifier in oil-in-emulsion through modification of the interfacial behavior between oil and water. Moreover, CH possesses very high positive surface charge, and it is finely dispersed due to the repulsion interaction of same charge [8,9]. On the other hand, a CH-based microcapsule shell successfully controlled the release of the core oil with time [10].

Another polysaccharide, carboxymethyl cellulose sodium salt (NaCMC), is a derivative of cellulose with good solubility in water. The derivative is obtained by substitution reaction of hydroxymethyl groups by hydrophobic carboxymethyl groups in position 2, 3, and 6. NaCMC has a linear polymer chain containing 1,4-linked glycans, and displays polyelectrolyte behavior due to weak acidic groups in the molecular backbone. The substitution degree, determined by the number (avg) of carboxymethyl groups per unit of NaCMC, is one of the major factors affecting the physicochemical behavior of NaCMC, which has many diversified applications, such as thickener, emulsion stabilizer, fat replacer in meat, binder, and film-forming properties [11,12]. Moreover, concerning the safety of human health and environment, NaCMC is biocompatible, biodegradable, and nontoxic [13].

Due to the growing demand of biobased materials for future sustainability, CH and NaCMC-based material and their applications will bring good future perspective in fundamental and applied aspects of new product formulations. Both CH and NaCMC have several properties which are affected by the change of pH, such as the swelling behavior, which allows increasing the matrix volume of PE and PEC upon changing the pH from acidic to basic conditions. This concept is applied in the encapsulation of active ingredients by hydrogel and subsequent release of these active components under favorable pH and ionic strength conditions. Therefore, it is worth studying the process parameters of PEC formation and PEC application in specific areas, such as microencapsulation. Several studies were carried out about the formulation of CH/CMC complex in the field of microcapsules [14,15], hydrogel [16,17], controlled drug delivery [18,19], etc.

The perspective applications of the polyelectrolyte complexes are required to be crosslinked by an efficient crosslinker to sustain the film in different processing conditions, such as high temperature or pH. Glutaraldehyde (GTA) is one of the most frequently used reagents for crosslinking polysaccharides and collagens. GTA is cheap, easily soluble in aqueous environment, and reacts very fast, although temperature, pH, and concentration affect the reaction rate. The crosslinking mechanism of GTA with collagen can be explained by two pathways; i.e., (1) the formation of Schiff bases between aldehyde and amino groups; (2) aldol condensation reaction between two aldehyde groups. However, GTA reacts with amino groups, including amido, carboxy hydroxyl, and other functional groups in the backbone of polysaccharides and proteins. Despite having dose-dependent skin irritation, prevalence of respiratory problems, no evidence of mortality, tumor evolution, carcinogenicity, genetic disorder and reproduction toxicity has been found in in vivo and in vitro studies of GTA [20]. Besides, no potential evidence of immunological reaction and interruption in healing process was observed after the impregnation of albumin and gelatin into Dacron grafts crosslinked by GTA [21,22]. In the evolution and proliferation of endothelial cells, GTA-crosslinked microcarriers were quite successful, but possible toxicity might arise upon release, due to degradation in the biological host cellular system [23,24]. On the one hand, genipin or epoxy materials have been considered as alternative crosslinking agents (although not as effective as GTA) while, on the other hand, GTA biocompatibility can be improved by minimizing the processing amount of GTA [25,26]. In contrast, a recent study found the inhibition of cell proliferation by GTA at 0.63 µg/mL which was equivalent to 50.0 µg/mL of genipin. The findings claimed that the GTA possessed 10 times higher cytotoxicity than that of genipin when the cell proliferation study was performed by using the cell line of L929 fibroblasts. Moreover, the MTT test by indirect extraction assay exhibited potential toxicity in fixed tissues [27].

Moreover, in the MTT test for cell viability using MTT solution; i.e., 3-(4,5-dimethylthiazol-2-yl)-2,5-diphenyltetrazolium bromide, the inhibition of MTT solution was exhibited by GTA-crosslinked collagen for dentin bonding, discouraging the use of GTA for clinical applications [28]. Another recent study exhibited that GTA-crosslinked chitosan–gelatin film exhibited non-toxicity and acceptable cell viability in the release of lupeol in wound healing applications [29]. Despite having some contradictory findings, the intention of using GTA in the present study was to observe the primary behavior of crosslinking, and the corresponding encapsulation property of the CH–NaCMC system, but in the context of developing a completely biobased product formulation, or plant extracted crosslinker; e.g., genipin from *Fusarium solani* is one of the best choices in the subsequent future study of the present project.

In this study, chitosan (CH) and carboxymethyl cellulose sodium salt (NaCMC) have been used as PEs to form CH–NaCMC-based PEC. The layer-by-layer technique was adopted to prepare PEC on the surface of emulsion droplets. PEs form stable emulsions without involvement of surfactants, especially when the polymers show good surface-active properties. In the present study, the emulsions were prepared by two subsequent steps. In the first one, CH-based emulsions were prepared at different pH values, and the morphology, size, and droplet surface charge were analyzed to optimize the conditions for stable emulsion formulation. The second step consisted in forming a saturated surface NaCMC layer on the emulsion droplets. In this stage, zeta potential measurements were carried out to confirm the interaction between CH-based emulsion droplets and NaCMC, and optical microscopy was used to verify the survival of emulsion droplets. The hypothesis of the study was that CH molecules adsorbed on the emulsion can interact with the oppositely charged NaCMC, and that a saturated bilayer can be obtained by complete coverage of the droplet surface. The oppositely charged layers can be applied on the surface of the emulsion to reinforce and tune the stability of the microcapsule formulation. Therefore, the main aim of the study is to investigate the fundamental mechanism of interaction between oppositely charged CH and NaCMC, and explore thermal properties and encapsulation efficiency of paraffin in the polysaccharide-based shell. The layer-by-layer technique was used for the formation of CH–NaCMC complex deposited on the paraffin emulsion droplets. The originality of the present study is the bilayer formation of CH and NaCMC, and the tunability of the oppositely charged layers (by increasing the number of layers) on emulsion droplets for desired future microcapsule applications, such as thermoregulating textile finishing (PCM).

## 2. Results and Discussion

### 2.1. Effect of pH on the Bulk Aqueous PE Solution

**Figure 1:**
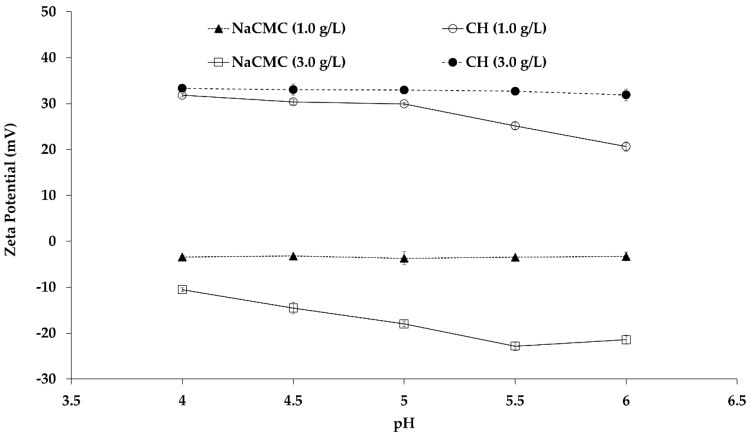
Zeta potential values of aqueous chitosan (CH) and carboxymethyl cellulose sodium salt (NaCMC) at different pH. Mean values with 95% confidence interval.

The properties of PE solutions (NaCMC and CH) depend on pH, CH degree of deacetylation (DD), NaCMC degree of substitution (DS), and PEs concentration. In the present study, the zeta potential values were measured as a function of pH and concentration. Figure 1 demonstrated the influence of pH on charge property of aqueous NaCMC and CH at concentrations of 1.0 and 3.0 g/L. The zeta potential measurement of aqueous NaCMC showed negative values in the entire range of experimental pH (4.0–6.0) indicating the NaCMC anionic behavior due to ionization of carboxylic groups in the NaCMC backbone. The literature reported that complete dissociation of NaCMC was observed at neutral pH 7.0 [12]. NaCMC solution at 1.0 g/L has a zeta potential of −3.5 mV, and the value was steady, irrespective of the pH. At a NaCMC concentration of 3 g/L, the zeta potential values decreased to −10 mV at pH 4.0 (compared to −3.5 mV at 1 g/L), and gradually dropped to more negative values up to −22.5 mV at pH 6.0. Basically, NaCMC aqueous solution acts as a polyelectrolyte because one polymer chain electrostatically attracts many different ions of opposite charge, i.e., Na^+^ and H^+^, depending on the availability of the oppositely charged groups, i.e., –OCH_2_COO^−^. Besides, the repulsive interaction between similar charges is maximized only when the polymer chain is fully extended in the solution. By contrast, general electrolytes (such as NaCl or KCl etc) in aqueous solution contain equal numbers of positive and negative ions, which move independently in the solution. Moreover, the polyelectrolyte solution is also different from the typical neutral polymer solution. The typical polymer chain conformation is random coil in a polymer solution, and the coiling is non-responsive upon changing the concentration, even though the solution is highly diluted in a good solvent. By contrast, the polyelectrolyte is fully ionized and dissociated at infinite dilute solution, and the polyelectrolyte chains exhibit a fully charged state. As a result, the chain is stretched or coil conformation depends on the charge repulsion of the PE backbone. NaCMC is a derivative of cellulose in which –CH_2_COOH functionalization takes place by the replacement of –OH groups from the cellulose backbone.

In highly acidic aqueous medium (pH < 3.5), the NaCMC polymer chain was very poorly ionized, and consequently very packed and highly dense [30]. When the pH was raised close to the p*K*a of CMC (around pH 3.8–4.0), segregation of the molecular chains occurred due to the presence of charged species, and led to considerable reduction of repulsive interactions. As a consequence, diffusion of counterions (Na^+^ and H^+^) around the CMC chain started due to the negatively charged –OCH_2_COO^−^ groups. Furthermore, those counterions were involved in a competitive process by two physical phenomena—condensation and free diffusion, depending on the pH. The condensed counterions bind strongly in the inner side of NaCMC backbone within a narrow potential zone while the diffused counterions move around the PE chain surrounding loosely and freely. At low concentrations of NaCMC (1.0 g/L), the condensation of Na^+^ (contributed to by NaCMC and sodium acetate) occurred in such a way that the intramolecular repulsive force was reduced, facilitating CMC chain coil formation, as confirmed by the reduction of intrinsic viscosity. Therefore, the pH increases from 4.0 to 7.0 in the aqueous NaCMC solution had no effect on the av zeta potential value at low concentration (1.0 g/L) [12], as two neighboring charges of polyelectrolyte chains exhibited a distance lower than 0.7 nm in this counterion-driven circumstance. When the concentration of NaCMC was increased, the oppositely charged counterions were drawn back to the polymer chain to neutralize the charge on the chain, resulting in a less extended and partially coiled conformation. This effect weakened the intramolecular repulsive force and progressively increased chain coiling with the increase of the concentration until the chain exhibited the undissociated and fully coiled structure. The coiled PE conformation affected the hydrodynamic radius of the polymer solution, and consequently, the mobility of the polymer segments, causing a higher zeta potential at high PE concentration. Therefore, upon increasing the concentration to 3.0 g/L, NaCMC molecular chain exhibited more negatively charged species with respect to 1.0 g/L NaCMC, which induced a progressive switch from the coil conformation to the expansion state, when the solution pH was close to p*K*a 4.0. As the pH increased gradually above p*K*a, the negatively charged species (–OCH_2_COO^−^) increased dramatically (complete dissociation occurs at neutral pH 7.0), and consequently, the intra- and intermolecular repulsion interactions increased, as demonstrated by the stronger zeta potential of −22.5 mV. At this stage, the intermolecular repulsive forces acted against the intramolecular repulsive interaction and expansion of NaCMC chain, causing deprotonation and formation of dissociated carboxymethyl species [31].

On the other hand, CH molecules in the aqueous solution played a role as polyelectrolyte containing functional groups, i.e., *N*-acetyl and amine groups. The hydrogen bonds are involved in the inter- and intramolecular interactions even before dissolution in an aqueous medium. There are two ways in which hydrogen bonds are formed: (1) Between the carbonyl containing acetyl groups and CH_2_OH groups; (2) between the hydroxyl groups and the deoxy of the neighboring molecules. In the present study, the DD value of 80% indicated a certain extent of protonation of the deacetylated units of CH, which induced the breaking of the existing hydrogen bonds by introducing interchain repulsions, and allowed dissolution [32]. Moreover, a very high dissolution of CH occurred in the aqueous buffer by increasing the polymer–solvent interactions in acidic condition (sodium acetate buffer pH 4.0). Therefore, a more extended conformation or hydrodynamic volume of CH molecules was observed due to incomplete neutralization of positive charges at the lower pH of 4.0. As a result, the zeta potential value around +31.9 and +33.3 mV were observed for both 1.0 and 3.0 g/L of CH concentration, respectively, suggesting that highly expanded CH chain conformation occurred by a few entanglements which allowed a stiffer structure. With a gradual increase of pH to higher values (up to pH 6.0), the high salt concentration in the solution allowed only weak solvent–CH interactions, resulting in a random coil structure. In other words, the polymer–polymer interactions were stronger at pH 6.0 than pH 4.0, and consequently, the structure was more compact in the aqueous medium. Therefore, the degree of protonation of amine groups decreased, and the positive zeta potential decreased from +31.9 mV to values around +20.0 mV for 1.0 g/L of CH concentration. By contrast, no change was observed in the positive zeta potential value as CH concentration increased to 3.0 g/L. This result suggests that pH had very little effect at 3.0 g/L CH concentration, as it was observed in the literature [9]. The hydrophobic interactions among the *N*-acetyl-d-glucosamine units dominate over the columbic repulsion of the protonated CH amino groups, resulting in intermolecular aggregation. Besides, the CH deacetylated groups molecules act both as proton donor and acceptor in aqueous medium, inducing formation of hydrogen bonds among the chains and contributing to intermolecular aggregation. As a result, a mutual contribution of hydrophobic and hydrophilic bonds played a major role in the intermolecular interactions and aggregation of CH molecules, with very little variation of the zeta potential, i.e., electrophoretic mobility was observed throughout the entire experimental pH (from 4.0 to 6.0) at 3.0 g/L [33].

### 2.2. Surface Adsorption Behaviour of CH and NaCMC

**Figure 2:**
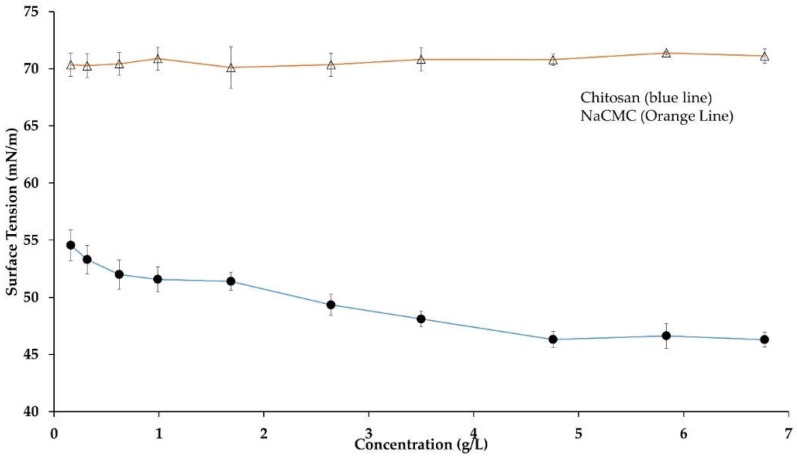
Surface tension of aqueous CH and NaCMC at pH 5.5 and 40 °C. Mean values with 95% confidence interval.

Surface adsorption is one of the important properties of amphiphilic molecules caused by the fact that the polar sites of the molecule tend to be exposed to water, and non-polar ones to air on the meniscus layer. In general, the disruption of cohesive force allows the amphiphilic molecules to be packed on the surface with a reduction of surface tension. In the present study, the surface tension of both aqueous CH and NaCMC were measured to know the surface active behavior (Figure 2). The NaCMC molecules exhibited an almost constant value of surface tension of approximately 70.0 mN/m, which was the same as the aqueous buffer. This result indicated that NaCMC molecules were not adsorbed at the air–water interface, and did not act as a surfactant at the experimental concentration, as confirmed by the literature [34].

On the other hand, surface tension decreased with the increase of aqueous CH concentration and reached a plateau value of 50.0 mN/m ± 3.5 mN/m above 3.0 g/L CH concentration at pH 5.5 and 40 °C after 24 h (Figure 2).The surface tension value was consistent with published works, where 2 g/L low molecular weight CH decreased water surface tension to 52.5 mN/m.The present study was fully consistent with the published study where Langmuir film balance technique was used to assess the surface activity of aqueous CH [35]. Moreover, CH contains hydrophilic deacetylated NH_2_ groups and hydrophobic *N*-acetyl groups (degree of acetylation, DA = 20%). The hydrophobic and hydrophilic moieties are randomly distributed along the CH backbone. Therefore, the surface tension reduction resulted from the combined effect of steric interference and columbic repulsion of CH-ionized moieties. The initial stage of adsorption at air–water surface occurred by diffusion-controlled mechanism from the bulk to the meniscus, and anchored the molecules in the first layer in contact with the air. In the subsequent step of the adsorption process, an adsorption layer was formed, which acted as a repulsive wall for the arriving molecules and decreased the surface tension over a long period [36]. However, apart from the hydrophobicity of the acetyl groups (or hydrophilic–lipophilic balance, HLB), the surface activity also depends on the intra- and interpolymer interactions. The amino groups of aqueous CH acted as proton donor and proton acceptor moieties, and influenced the intermolecular aggregation to enhance surface activity [33]. The adsorbed CH monolayer occupies a surface around 3.88 × 10^−6^ mol/m^2^, so that a single CH molecule and a glucosamine unit of CH backbone accounts for around 0.5 nm^2^ and 0.43 nm^2^, respectively [35].

### 2.3. Emulsion Preparation

As mentioned above, the emulsion was prepared in two steps for complex layer formation by two oppositely charged PEs. Emulsion-1 was CH-based, since CH provided the surface adsorption property. The optimal emulsion formulation was chosen by considering the following aspects: (1) A single emulsion with mean droplets of size less than 5.0 µm were formed, and (2) the pH allowed CH to display charge properties (to exert stabilizing repulsive forces). Emulsion-2 was prepared by forming an additional layer on the droplets of Emulsion-1 by adding NaCMC solution and optimizing NaCMC concentration to achieve a saturated bilayer.

#### 2.3.1. Influence of pH on CH-Based Emulsion-1 Droplets

**Figure 3:**
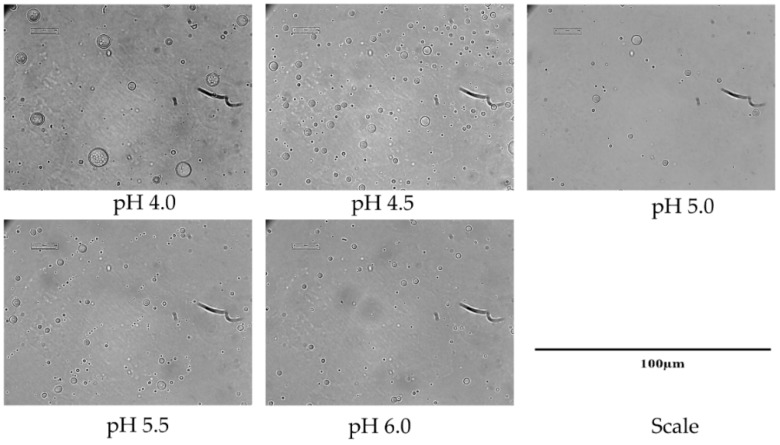
CH-based droplets (Emulsion-1) at different pH.

**Figure 4:**
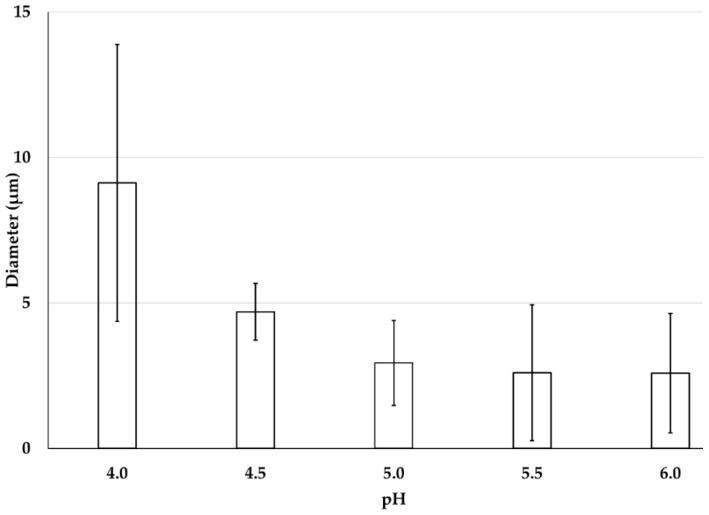
Mean diameter of CH-based Emulsion-1 at pH from 4.0 to 6.0. Mean values with 95% confidence interval.

Five different formulations were considered for the preparation of Emulsion-1 by varying the pH from 4.0 to 6.0 at 40 °C. The size and surface charge of emulsion droplets were determined by microscopic images, mean diameter calculation, and zeta potential. When the emulsion was prepared at the lowest pH 4.0, a water–oil–water (W/O/W) or double emulsion was observed (black circle entrapped by a large droplet in Figure 3). In this case, droplets of dispersed oil phase were encapsulated in a very tiny amount of continuous water phase trapped inside the droplets. The double emulsion was stable in the continuous phase. The reason behind the formation of the double emulsion can be explained by the combined effect of DD of CH molecules and the conformational structure of CH at this specific pH. The double emulsion formation can be explained by two reasons: (1) CH molecules exhibited stiff conformation at pH 4.0 or lower; (2) on average, 20% hydrophobic sites were available in the CH structure. Low DD values facilitated water-in-oil emulsions, while high DD values promoted oil-in-water emulsions, since different DD values are associated with a different hydrophobic–hydrophilic balance [20]. Two phenomena occurred at acidic pH 4.0—the CH coil-like conformation was extended, and it was highly protonated, resulting in a stiff conformation. Therefore, steric hindrance dominated due to the bulky acetamido groups resisting rotation around the glycosidic bonds, and the hydrophobic sites of CH were not able to anchor in the oil phase, due to CH rigidity. Consequently, the W/O/W emulsion was formed after the subsequent formation of W/O emulsion. Upon increasing the pH (up to 6.0), single emulsion droplets were observed by microscopic analysis, indicating that the thermodynamically unstable double emulsion disappeared, probably thanks to a more flexible CH chain conformation at higher pH (4.0 to 6.0) [21]. Alternatively, the evaporation of the inner aqueous phase took place, resulting in a collapse of the whole emulsion droplets. The observation is consistent with the average droplet size of emulsion displayed in Figure 4. The droplet size or mean diameter of emulsion was around 9.0 µm at pH 4.0 and 2.6 µm at pH 6.0, showing a decreasing trend as the pH increased. The zeta potential values displayed a decreasing trend upon increasing pH, since CH molecules were deprotonated on the emulsion droplets [3,37]. The emulsion at pH 5.5 was considered as an effective one because small droplet sizes were achieved with a moderate zeta potential of +25.2 mV, without CH precipitation due to the complete deprotonation that occurred at pH ≥ 6.0. It is important to note that the zeta potential measurement with colloidal emulsion should be carried out with special care due to the aggregated and viscous form of Emulsion-1. Therefore, the measurements were carried after the dilution of Emulsion-1 at 1:10 in the same buffer medium, and the final CH concentration was 10 × 10^−2^ g/L.

#### 2.3.2. Influence of NaCMC Concentration on Emulsion-2

**Figure 5:**
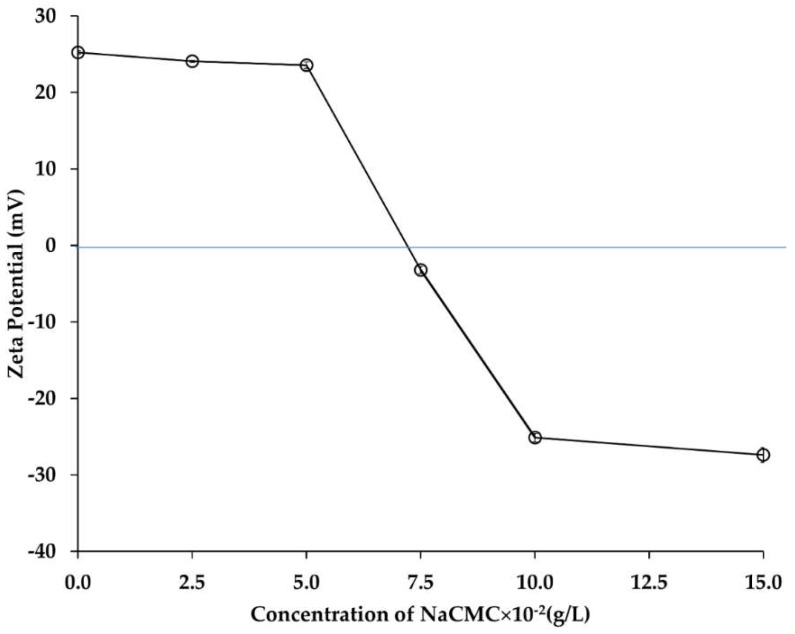
Zeta potential values after NaCMC layer formation in Emulsion-2. The blue line indicated the neutral (charge balanced) zone of zeta potential. Mean values with 95% confidence interval.

The main intention of this phase was to identify the influential factors in formulating a second layer of NaCMC on CH-based Emulsion-1. In this case, the optical images, and zeta potential measurements of Emulsion-2 were determined at different concentration of NaCMC. In the absence of NaCMC, the zeta potential value of the diluted Emulsion-1 (CH concentration 0.1 g/L and pH 5.5) was +25.2 mV. Upon addition of negatively charged NaCMC, the positive charge of the droplet surface became less positive, and then tuned from positive to negative as NaCMC concentration was increased. The addition of aqueous NaCMC concentration between 2.5 × 10^−2^ g/L and 15 × 10^−2^ g/L decreased the ξ value from +25.2 mV to −27.4 mV, respectively (Figure 5). When the concentration of NaCMC increased to 7.5 × 10^−2^ g/L, the zeta potential value switched from positive to negative (−3.2 mV). This suggested that complex formation occurred between NaCMC and CH on the surface of Emulsion-1 droplets. With further increase of NaCMC concentration to 10 × 10^−2^ g/L, the zeta potential value exhibited a stable negative value (−25 mV), and no significant change was observed when the concentration of NaCMC reached 15 × 10^−2^ g/L. This result suggested that the saturation level of CH–NaCMC complexes onto the surface of droplets was achieved. Similar behaviors were claimed between anionic polysaccharide and positively charged protein in a previous study [38,39,40]. NaCMC is a negatively charged polysaccharide exhibiting negative ξ values in the studied pH range (Figure 1) [38]. The reduction of the positive ξ values was due to neutralization of the positively charged CH by the negatively charged NaCMC that occurred on the surface of the Emulsion-1 droplets. Upon gradual increase of NaCMC concentration (2.5 × 10^−2^ g/L to 15 × 10^−2^ g/L), the surface of Emulsion-1 droplets became saturated with the negatively charged NaCMC, by adsorbing on the positively charged CH and forming a polyelectrolyte complex layer. The interaction between two oppositely charged polyelectrolytes and subsequent layer formation mechanism can be explained based on base of the layer-by-layer (LBL) assembly. When NaCMC molecules dissolve in an aqueous solution, the diffusion of counterions (Na^+^ and H^+^) around the NaCMC chain occurred due to the presence of negatively charged groups, COO^−^. Consequently, an electric double layer is developed around the polyelectrolyte chains, limiting the mobility of the counterions which are localized in the proximity of the ionic part of the PE chains. Upon mixing NaCMC solution with the oppositely charged CH-based Emulsion-1, the electric double layer was destroyed, and the counterions released with increase of entropy. This circumstance led to an overall decrease of Gibbs free energy, determining the formation of a stable complexation, compromising dilution entropy and coulombic attraction. The gain in entropy due to the liberation of counterions compensated the translational entropy loss of NaCMC chain upon complexation with CH. Therefore, CH–NaCMC interactions were more dominant over the interaction between individual polyelectrolyte (CH or NaCMC) and their counterions [41]. Emulsion-2 formation can be split in three different stages: firstly, electrostatic interactions between oppositely charged NH_3_^+^ and COO^−^ of CH and NaCMC, respectively, were established at around 7.5 × 10^−2^ g/L NaCMC (Figure 5); secondly, the CH and NaCMC were rearranged thanks to the loss of configurational freedom that occurred in the range 7.5 × 10^−2^–10 × 10^−2^ g/L NaCMC, and thirdly, hydrophobic interactions through the aggregation of CH and NaCMC backbones occurred above 10 × 10^−2^ g/L NaCMC. Interestingly, the ratio of CH and NaCMC in the complex layer in Emulsion-2 was found to be 1:1 (both PEs contained at 10 × 10^−2^ g/L). The polyelectrolyte complex formation was optimized by forming a saturated layer of NaCMC on CH-based Emulsion-1 at pH 5.5 with a corresponding mass ratio of 1:1. The crosslinking of the complex layer was carried out for the above-optimized formulation, and described in the next section.

### 2.4. CH–NaCMC-Based Microcapsule Shell Formation

#### 2.4.1. Mechanism of Shell Formation

**Figure 6:**
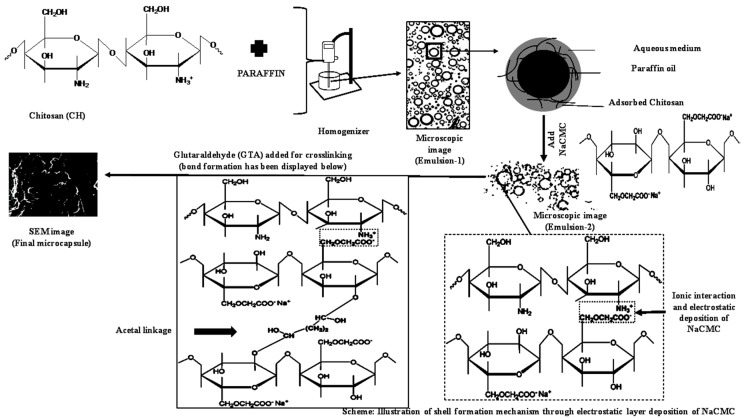
Mechanism of CH–NaCMC complex and crosslinking shell formation.

A schematic figure of the process, including shell formation mechanism, was displayed in Figure 6. The outer layer of Emulsion-2 prepared with CH/NaCMC ratio 1:1 was crosslinked using 25% *w*/*v* % GTA to form a hard shell around paraffin oil. Primarily, ionic interaction forces acted for the preparation of a stable polyelectrolyte complex with the domination of negatively charged surface. In the next step, GTA formed covalent bonds with the existing –OH groups of NaCMC and –CHO groups of GTA in the polyelectrolyte complex around the paraffin. GTA, with two aldehyde groups at its ends, allowed very prompt reaction with hydroxyl groups. Therefore, the polyelectrolyte complex was turned into a hard layer around the liquid paraffin, whose strength was due to (1) ionic interactions and (2) formation of acetal (–C–O–C–O–C–) linkages.

#### 2.4.2. Structure of the Microcapsule Shell

**Figure 7:**
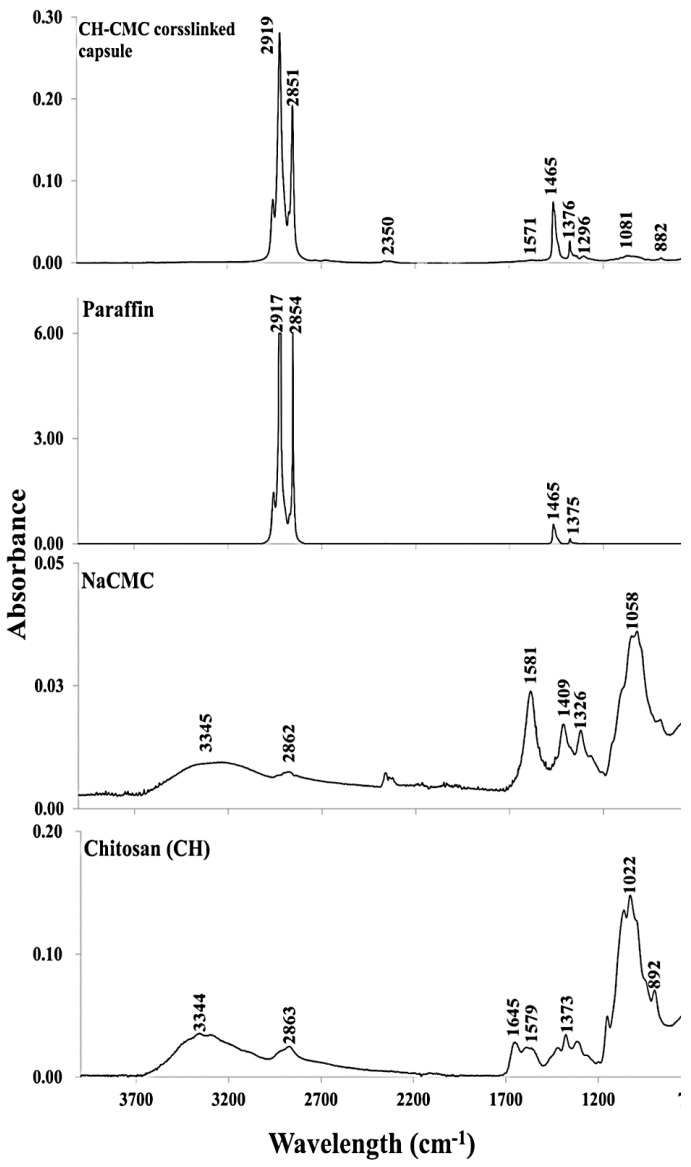
ATR-FTIR analysis of pure CH, NaCMC, paraffin oil, and crosslinked microcapsules.

The ATR-FTIR spectra of paraffin, CH, NaCMC, and crosslinked CH–NaCMC mixture and microcapsules, were evaluated and displayed in Figure 7. The presence of O–H and N–H bonds of primary amine were observed by generating a wide absorbance area in the range of 3400–3000 cm^−1^ in the absorption spectrum of CH. Another broad area of absorption spectrum in the range of 3000–2800 cm^−1^ was due to H–C–H and C–H groups of the pyranose ring of polysaccharide. In both cases, overlaps of functional groups’ absorption bands were observed broadly from 3350 cm^−1^ to 2700 cm^−1^ for pure CH molecules. The absorption peaks in the range from 1700 cm^−1^ to 1480 cm^−1^ identified the carbonyl functional groups derived from amide II at around 1645 cm^−1^, and the vibrational frequency of the amine group at around 1579 cm^−1^. Moreover, CH_3_ and CH_2_ exhibited absorption bands between 1450 cm^−1^ to 1350 cm^−1^. Some additional bonds, such as CO, C–O–H, C–O–C, and CH groups in CH molecular backbone, were displayed in the range of 1360 cm^−1^ to 800 cm^−1^ [9].

The spectrum of pure NaCMC shows a broad spectrum at 3345 cm^−1^ due to the presence of –OH groups in a stretched condition. The absorption band at 2862 cm^−1^ was ascribed to the C–H stretching vibration of the pyranose ring. Moreover, the strong absorption spectrum at 1581 cm^−1^ indicated the COO^−^ groups in the CMC backbone. Two peaks at 1409 cm^−1^ and 1326 cm^−1^ were attributed to the scission of CH_2_ and vibrational frequency of –OH bonds, respectively. Another broad peak was observed at 1058 cm^−1^, which was mainly assigned to the stretching of H_2_C–O–CH_2_ bond of NaCMC backbone [42].

The absorption peaks of paraffin oil at 2917 cm^−1^ and 2854 cm^−1^ were due to the stretching and vibration of C–H bonds of *n*-alkane. The bending vibration of C–H bonds originated from CH_2_ and CH_3_, and were displayed by the peaks at 1465, 1375, and 717 cm^−1^ [9].

The crosslinking reaction between CH and NaCMC complex carried out by GTA (25% *w*/*v* aqueous solution) consolidated the microcapsule shell. The absorption spectrum of crosslinked microcapsules highlighted that the changes occurred after the crosslinking reaction with respect to pure NaCMC, CH, and paraffin oil functional groups. In general, GTA-crosslinked microcapsules did not exhibit absorption in the range of 3400–3000 cm^−1^, which indicated that –OH groups were absent in the microcapsule shell after crosslinking. Two strong absorption spectra were observed, similar to paraffin at 2919 and 2851 cm^−1^, attributed to the C–H stretching vibration of *n*-alkane. The low intensity peak at 1571 cm^−1^ was assigned to the ionic interaction between COO^−^ (displayed at 1581 cm^−1^ in NaCMC backbone) and NH_3_^+^ (representing spectrum at 1579 cm^−1^ in the CH skeleton). The absorption peak in the microcapsule spectrum indicated the complex coacervate formation between two oppositely charged polysaccharides. The bending vibration of methyl and methylene groups were observed at 1469 cm^−1^ and 1376 cm^−1^ for paraffin oil. However, the absorption bands in the range of 1320–1000 cm^−1^ were attributed to the stretching of C–O–C linkage which indicated the acetal bond formed by the reaction between hydroxyl and aldehyde groups provided by NaCMC and GTA, respectively [43,44,45]. It was confirmed that the outer layer sphere of the microcapsule was mainly covered by the adsorbed NaCMC whose hydroxyl groups were available to form acetal bonds. The peak at 882 cm^−1^ was observed for the hemiacetal or hydrated form after the initial reaction between aldehyde and hydroxyl groups of GTA and NaCMC [46].

#### 2.4.3. Morphology of the Microcapsules

**Figure 8:**
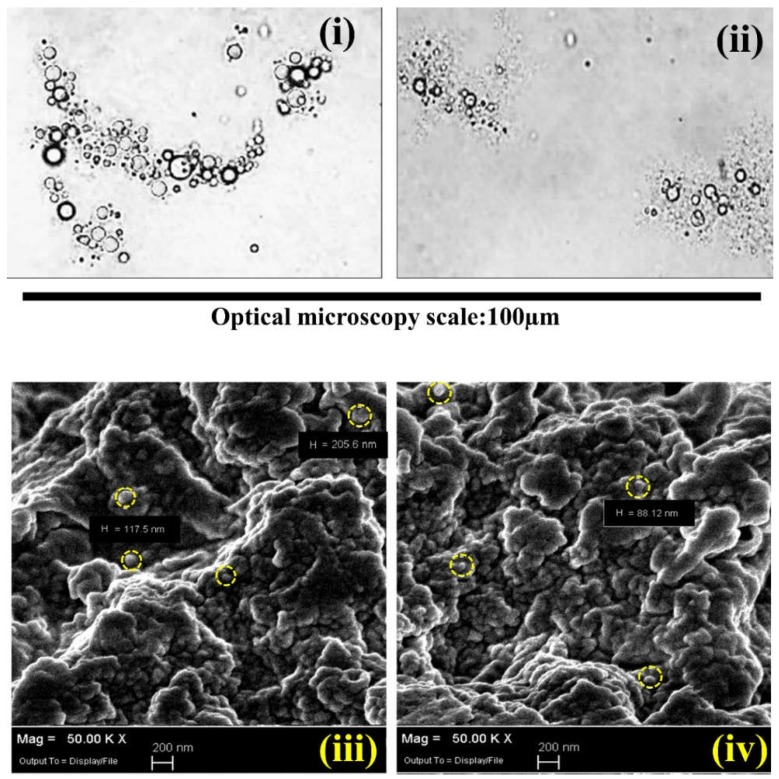
Optical microscopic images of crosslinked microcapsules with GTA. (**i**) 0.3 g and (**ii**) 0.6 g GTA per unit gram (g) of total biopolymer; SEM images of crosslinked microcapsules with (**iii**) 0.3 g GTA and (**iv**) 0.6 g per unit gram of total biopolymer. “H” indicates the diameter in nanometer unit (nm); the yellow circles indicate the typical spherical microcapsules in the aggregates.

The optical microscopic images were exhibited at 40 times bigger than the original size before drying (Figure 8i,ii), while the SEM image analyses were conducted after drying the microcapsules at 50K times higher resolution than the original size (Figure 8iii,iv). Both images of two microcapsule batches were obtained with different GTA/PE ratios: 0.3 g GTA (Figure 8i,iii) and 0.6 g GTA (Figure 8ii,iv) per g of total biopolymers. It was clearly observed that the microcapsules were aggregated in spherical or semi-spherical shapes of nanometer size. Some aggregations were fused together and displayed as a combined pile of polymers, probably due to the incomplete microencapsulation or shell materials crosslinked without properly encapsulated paraffin oil. The facility for the SEM analysis was not available after the preparation of microcapsules. Therefore, the SEM images of the microcapsules were taken 400 days after the microcapsule preparation and drying. During this time period, the microcapsules were kept in an air-tight plastic bag at 25 °C in the lab. The SEM analysis exhibited an aggregated form of spherical microcapsules after 400 days, which provided good stability under a controlled environment in the laboratory. Moreover, the microcapsules were crushed to check the encapsulated liquid paraffin in the microcapsules after 400 days. The availability of liquid paraffin was observed in the crushed microcapsules even after 400 days of microcapsule preparation.

#### 2.4.4. Effect of GTA on Microcapsule Thermal Properties

The DSC analysis was carried out to study the latent heat of melting and crystallization for the paraffin oil and encapsulated paraffin oil displayed in the Table 1. The peak melting temperature and onset melting temperature were observed at 28.1 °C and 20.0 °C, respectively, for pure paraffin oil, and the corresponding latent heat was 232 J g^−1^. The melting enthalpies of two samples were found to be 136.1 J g^−1^ and 155.5 J g^−1^ for 0.3 g and 0.6 g GTA per gram of total mass of PEs (CH and NaCMC), respectively, even though the amount of biopolymer ratio and *CT*% from Equation (3) was fixed. Even though the GTA amount did not have any contribution to melting enthalpy, the corresponding *CA*% (Equation (2)), encapsulation efficiency (*EE*%), and yield (*EY*%) values were increased. This suggested that the crosslinking reaction made the shell material more compact when higher amounts of GTA were added. Probably, the pores and core material leakage were reduced and, as a consequence, the surface to core ratio *CA*%, *EE*%, and *EY*%. A contrasting result was observed in the previous study [47]. The main difference between the previously published and present study was the result of *EE*% and *EY*%. It can be observed, clearly, in the previous study that the encapsulation efficiency was decreasing with increasing amounts of crosslinker GTA. By contrast, the present work claimed that the increased amount of glutaraldehyde increased the *EE*% (Table 1). The present work exhibited an improvement of the encapsulation efficiency by changing the whole formulation and repeating the characterization process. The microencapsulation system displayed a strong influence of the formulation parameter pH in the crosslinking step. In the previous study, the increase of emulsion pH above 6.0 was maintained before addition of GTA, which disassembled the polyelectrolyte complex due to the lack of electrostatic interactions [44]. Consequently, the crosslinking occurred only with the functional groups of the individual polymer, separately, which ultimately interrupted the shell formation and decreased the encapsulation efficiency with the increase of GTA. It is very important to maintain the pH below 6.0, especially when the polyelectrolyte complex contains a CH–NaCMC system, and allows the crosslinking reaction with the polyelectrolyte complex by avoiding any repulsion between CH and NaCMC. Therefore, the present work exhibited the influence of GTA on the microencapsulation process by increasing the encapsulation efficiency. Moreover, FTIR results did not support the formation of covalent linkages by the crosslinking reaction in the previous study, and the fundamental discussion of polyelectrolyte complexation and interaction was not properly explained, which has been discussed in depth in the present study. On the other hand, the melting temperature decreased with the increase of GTA concentration, and this result can be related to the microcapsule morphology. SEM analysis showed the size of the microcapsules in the range of 80 nm to 200 nm, which determined the limited mobility of paraffin oil chains. It should be noted that the optical microscopic analysis expressed the mean size of the droplets, which were ten times bigger than the individual capsule compared to the SEM size. Consequently, the crystallinity of paraffin oil decreased, due to inhibited motion, and the melting temperature eventually decreased [31].

## 3. Experimental

### 3.1. Materials

NaCMC of *M_W_* ~90,000 Da and degree of substitution 70% (0.7 carboxymethyl groups per anhydroglucose unit) was purchased from Sigma-Aldrich France (Lyon, France). Low molecular weight CH (*M_W_* ~50,000–190,000 Da, DD 80% av) was purchased from Sigma-Aldrich France. Paraffin oil was purchased from SESOL Performance Company (Hamburg, Germany). Analytical grade glutaraldehyde, sodium acetate, and acetic acid were purchased from Sigma-Aldrich.

### 3.2. Solution Preparation

Sodium acetate and acetic acid were used for the preparation of the buffer solutions. First of all, 1.0 M stock solutions of sodium acetate and acetic acid were prepared separately. The stock solutions were diluted 1:5 with milliQ water to obtain two 0.2 M sodium acetate and acetic acid solutions in separate beakers. Five different buffer solutions, from pH 4.0 to pH 6.0, were prepared by mixing the 0.2 M sodium acetate and 0.2 M acetic acid solutions at an appropriate ratio. Five aqueous CH and NaCMC solutions were prepared by dissolving, respectively, 1.0 g and 3.0 g of CH and NaCMC in 100 mL of each buffer under magnetic stirring at 500 rpm for 24 h. As CH is hardly soluble at pH 6.0, the CH solution was prepared at buffer pH 5.5, and then the pH was adjusted to pH 6.0 by adding sodium acetate solution. All solutions were diluted with the corresponding buffer solutions to maintain the concentrations of each PE at 1.0 g/L and 3.0 g/L for zeta potential measurements. The zeta potential values of aqueous NaCMC and CH solutions were measured in the pH range from 4.0 to 6.0. Below its p*K*a of 3.6, NaCMC exhibits negligible dissociation and a packed dense structure due to lack of intramolecular charge repulsion, which results in poor solubility. Therefore, the experimental initial pH was chosen above NaCMC p*K*a (pH 4.0) and within pH 6.0, due to poor CH solubility above this pH.

### 3.3. Emulsion Preparation

The emulsion was prepared in two subsequent steps and named as Emulsion-1 (after the first step) and Emulsion-2 (after the second step). Emulsion-1 was prepared at pH 4.0 by dosing CH (1.0 g), paraffin oil (20.0 mL), and buffer solution (approximately 85.0 mL to make up to 100.0 mL mark). Firstly, the prepared CH solution of pH 4.0 was poured in the homogenization beaker; then, paraffin oil was added dropwise at the initial speed of 5000 rpm by using the ULTRA-TURRAX T-25 homogenizer (IKA, Staufen, Germany) for two minutes. The speed of the homogenizer was increased to 13,500 rpm after paraffin oil was entirely added to the homogenization beaker and stirring continued for 30 min. The homogenization time and speed were optimized to achieve emulsion droplets of size <5.0 µm [9]. The samples were collected for optical microscopy and zeta potential analysis after dilution 1:100 in pH 4.0 buffer medium. Different emulsion-1 types were prepared by using the other buffer media, i.e., 4.5, 5.0, 5.5, and 6.0 with the same procedure. Further analysis was carried out to optimize the best formulation by measurement of droplet size and single emulsion formation. The emulsion produced at pH 5.5 was identified as the optimal Emulsion-1 in terms of minimum droplet diameter.

After identification of the process conditions for obtaining the optimal Emulsion-1, different Emulsion-2 types were prepared starting from Emulsion-1, according to the following procedure: Emulsion-1 was diluted in pH 5.5 acetate buffer under magnetic stirring at 500 rpm and distributed in five beakers uniformly to prepare Emulsion-2. In the dilute emulsion, the CH concentration (10 × 10^−2^ g/L) was calculated based on the initial amount of CH used in Emulsion-1 considering that the homogenization at 13,500 rpm formed a uniformly distributed CH-based emulsion.

A stock solution of 5 mg/mL NaCMC was prepared separately in pH 5.5 buffer medium. Five different volumes, i.e., 0.5, 1.0, 1.5, 2.0, and 3.0 mL of the NaCMC stock solution were added dropwise in all five beakers of diluted Emulsion-1 separately. The zeta potential measurement of the new emulsion (known as Emulsion-2) was carried out after each volume addition. Five different volumes of NaCMC were chosen for the formulation of Emulsion-2 as the zeta potential values achieved a constant value (approximately). The NaCMC concentration range was 2.5 × 10^−2^ g/L to 15 × 10^−2^ g/L.

### 3.4. Microcapsule Preparation

Microcapsule preparation was carried out by crosslinking reaction of Emulsion-2 with glutaraldehyde (GTA 25% *w*/*v*). The crosslinked PEC acted as shell around the core of paraffin microcapsule. The preparation was conducted as followed: two different GTA aqueous concentrations were chosen and tested (0.3 g and 0.6 g per unit g of total biopolymers). Before adding GTA, Emulsion-2 was stirred for one hour to disperse the emulsion droplets in pH 5.5 buffer. The required amount of GTA solution was mixed with pH 5.5 buffer to maintain the abovementioned GTA/biopolymer ratio. When GTA solution was added dropwise to Emulsion-2, the emulsion turned into a yellowish suspension (from white). The crosslinked microcapsules slowly went up to the air–water interphase because of paraffin specific gravity, around 0.75. Then, the reaction vessel was transferred to an ice bath to maintain the temperature below 10 °C overnight. After crosslinking, the microcapsules were filtered and rinsed thoroughly with 0.1 M NaOH solution on a magnetic stirrer at 250 rpm for 60 min. The microcapsule suspension was then centrifuged at 300 rpm for 5 min. The excess CH and GTA were removed by rinsing the microcapsules in 100 mL water three times. The microcapsules were collected after centrifugation at 300 rpm for 5 min and rinsed with ethanol for two times on filter paper (Whatman^™^ 41 µm, Sigma-Aldrich) for removing any unencapsulated paraffin oil. Moreover, as ethanol also interacts with the active amino groups of chitosan, the formation of any kind of salt with CH was avoided. No changes in the CH structure occurred in the final microcapsules thanks to ethanol washing. Finally, the microcapsules were dried in the oven at 90 °C for 1 h to remove excess ethanol and moisture. It is worth mentioning that no variation in the pH should occur during GTA crosslinking reaction. The pH increase may disassemble the polyelectrolyte complex layer deposited on the emulsion droplets, affecting encapsulation efficiency by leaching of the core material in the reaction medium.

## 4. Characterization

### 4.1. Zeta Potential

The charge property of PE and PEC was measured by Zeta Sizer 2000 (Malvern Instrument, Worcestershire, UK). The emulsions were diluted 1:200 in the same buffer before measurements. All measurements were done in triplicate.

### 4.2. Surface Tension

The surface tension measurements were carried out by Wilhelmy’s method using a GBX 3S tensiometer (GBX, Romans sur Isere, France). The platinum plate for measuring the surface tension was flame-cleaned before analysis to avoid any contamination of the liquid surface. The glass vessel was cleaned with chromic-sulfuric acid solution, rinsed with Milli-Q water, and dried. The sequence of surface tension measurements started from a very diluted PE (CH or NaCMC) solution and, by subsequent additions, the concentration was increased until the surface tension become constant. This indicated a saturation of surface tension, namely, the condition when the meniscus was fully packed by the biopolymers. The equilibrium value of surface tension was recorded by taking several readings from the starting concentration of 0.07 g/L to 7.00 g/L at 120-min intervals, until a stable value was observed. The standard deviation of these readings was within ±0.4 mN/m. The detailed procedure of the experiment can be found elsewhere [35].

### 4.3. Microcapsule Morphology

#### 4.3.1. Optical Microscopy

The micrographs of the emulsions prepared at different pH (4.0 to 6.0) were recorded by an optical microscope (Axioskos Ziess, Thornwood, NY, USA) associated with a CCD camera (uEye, Obersulm, Germany) and programmed software (Perfect Image, Perfect Image 12, Newcastle, UK) at 40× magnification. One drop of emulsion was deposited on a glass slide, and checked directly under the microscope. In the case of a very concentrated emulsion, it was diluted 1:200 in the same buffer solution to avoid any effect due to overlapping of microcapsules. All images were analyzed by the imaging processing software ImageJ (Public domain, https://imagej.nih.gov/ij/) to determine the emulsion droplets’ diameter.

#### 4.3.2. SEM Image Analysis

The microcapsules were analyzed by scanning electron microscopy (Gemini SEM, Carl Zeiss, Oberkochen, Germany). Images of different resolutions were captured (30 k and 50 k times) by maintaining 5 kV voltage. The sample was fixed on the aluminum step by Ni–Cu conductive tape. All samples were metalized with chromium by sputtering technique (a Balzers Union Sputter apparatus, Berlin, Germany).

### 4.4. ATR Analysis

The ATR-FTIR analysis was carried out employing diamond ATR device on a spectrophotometer model of Nicolet FTIR 5700 (Thermo Fisher Scientific, Waltham, MA, USA). The spectra were collected by a resolution of 4 cm^−1^ and total of 128 scans for the samples of pure CH, pure NaCMC, pure paraffin oil, and the paraffin encapsulated CH–NaCMC microcapsules.

### 4.5. Differential Scanning Calorimetry (DSC)

The thermal responses of paraffin oil and encapsulated paraffin oil were recorded using the Instruments TGA/DSC 3+ (Mettler Toledo, Viroflay, France). The temperature was calibrated using indium under a fixed nitrogen stream (50 mL/min). All samples, of weight around 10 mg, were placed in the aluminum pans, and sealed before placing in the calorimeter. The scanned temperature range was fixed between −20 °C to 60 °C with an increment of 3 °C/min. After reaching 60 °C, the sample was maintained for 10 min to remove any thermal records, and was then cooled at 3 °C/min from 60 °C to −20°C. The final second cycle, between −20 °C to 60 °C at 3 °C/min, was considered to determine the melting isotherm and crystallization exotherm during cooling.

### 4.6. Characterization of Microcapsule

#### 4.6.1. Encapsulation Yield (*EY*%)

The encapsulation yield was calculated from the ratio between the mass of dry microcapsules (*m*_mic−cap_) and the total mass (*m*_t_) of the components (biopolymers and paraffin). It can be expressed by Equation (1).

(1) EY % = (mmic−cap mt) × 100

#### 4.6.2. Content of Paraffin Oil (*CA* %)

The content of paraffin oil is the practical amount of paraffin oil successfully encapsulated by the CH–CMC shell. It was determined from the ratio between the enthalpy (Δ*H*_mic−cap_) of the microcapsule and the enthalpy (Δ*H*_p_) of pure paraffin oil. The relation can be expressed by Equation (2).

(2) $$CA\;\%\;=\;\left(\frac{\Delta H_{\mathrm{mic}-\mathrm{cap}\;}}{\Delta H_{p}}\right)\;\times 100$$

The theoretical amount of paraffin was calculated from the ratio between the mass of paraffin oil (*m*_p_) used in the experiment and the mass of all components *m*_t_. It can be expressed by the Equation (3).

(3) CT % = (mp mt) × 100

#### 4.6.3. Encapsulation Efficiency (*EE* %)

The *EE* % was achieved from the ratio between *CA* % and *CT* % by Equation (4).

(4) EE % = (CA CT) × 100

## 5. Conclusions

A CH-based oil-in-water single emulsion was successfully prepared at pH 5.5. The smallest stable emulsion droplets were, on average, around 2.6 µm. A multiple emulsion was observed at lower pH (4.0) with higher larger droplets of mean size approximately 9.0 µm. By adding an oppositely charged NaCMC solution, a complex was formed on the CH-based emulsion through electrostatic deposition followed by bilayer/multilayer formation. The saturation condition when NaCMC fully complexed the underlying CH-layer was observed with CH/NaCMC mass ratio 1:1. GTA allowed the crosslinking reaction on the outer surface of the emulsion, as proven by the acetal linkage formation. The effect of GTA concentration was investigated, and it was observed that the higher values of core content, encapsulation efficiency, and yield were obtained by increasing the amount of GTA. This result suggested the formation of a very packed and aggregated shell material around the encapsulated core. The study provides the basis for tuning the multilayer layer formation through electrostatic deposition around the microcapsules, which, in turn, could allow a controlled release of core material.

## Figures and Tables

**Table 1:** Thermal properties of CH–NaCMC-based microcapsules.

Sample Label	Tm Onset (°C)	Tm Peak (°C)	ΔHm (J/g)	Tc Onset (°C)	Tc Peak (°C)	ΔHc (J/g)	*CA*%	*EE*%	*EY*%
Paraffin oil	20.0	28.1	232.0	15.0	7.6	230.1	-	-	-
CH_1_-CMC_1_-GTA_0.3_	17.9	29.1	136.1	16.5	8.4	136.3	58.6	66.4	34.4
CH_1_-CMC_1_-GTA_0.6_	13.5	26.0	155.5	15.2	3.8	154.8	67.0	76.0	44.7

CH_i_-CMC_j_-GTA_k_: where i indicated the amount (*wt* %) of CH in the sample and j was assigned for the amount (*wt* %) of NaCMC, and k was the amount of GTA (g of GTA/g of total biopolymer). CA, EE, and EY indicated practical amount of paraffin oil, encapsulation efficiency, and yield, respectively. The dashes in the second column indicated that no calculation was carried out because it expressed the parameters of pure unencapsulated core material.
